# Rho Kinase's Role in Myosin Recruitment to the Equatorial Cortex of Mitotic *Drosophila* S2 Cells Is for Myosin Regulatory Light Chain Phosphorylation

**DOI:** 10.1371/journal.pone.0000131

**Published:** 2006-12-27

**Authors:** Sara O. Dean, James A. Spudich

**Affiliations:** Department of Biochemistry, Stanford University, Stanford, California, United States of America; The University of Birmingham, United Kingdom

## Abstract

**Background:**

Myosin II recruitment to the equatorial cortex is one of the earliest events in establishment of the cytokinetic contractile ring. In *Drosophila* S2 cells, we previously showed that myosin II is recruited to the furrow independently of F-actin, and that Rho1 and Rok are essential for this recruitment [1]. Rok phosphorylates several cellular proteins, including the myosin regulatory light chain (RLC).

**Methodology/Principal Findings:**

Here we express phosphorylation state mimic constructs of the RLC in S2 cells to examine the role of RLC phosphorylation involving Rok in the localization of myosin. Phosphorylation of the RLC is required for myosin localization to the equatorial cortex during mitosis, and the essential role of Rok in this localization and for cytokinesis is to maintain phosphorylation of the RLC. The ability to regulate the RLC phosphorylation state spatio-temporally is not essential for the myosin localization. Furthermore, the essential role of Citron in cytokinesis is not phosphorylation of the RLC.

**Conclusions/Significance:**

We conclude that the Rho1 pathway leading to myosin localization to the future cytokinetic furrow is relatively straightforward, where only Rok is needed, and it is only needed to maintain phosphorylation of the myosin RLC.

## Introduction

Cytokinesis involves the formation of a myosin II containing contractile ring at the furrow of dividing cells. The placement of this contractile ring is controlled by the small GTPase Rho1/RhoA [2], [3] which stimulates both actin filament formation at the furrow by localized activation of formin proteins [4] and ring contraction by activating Rho kinase (Rok) and Citron kinase, which can phosphorylate the myosin II regulatory light chain (RLC) [5]–[7]. Rok directly phosphorylates myosin II RLC at threonine 18 and serine 19 in mammalian cells [8] (T20 and S21 in *Drosophila* [9]) and suppresses its dephosphorylation by inactivating the myosin phosphatase [10]. Phosphorylation at these sites has been shown *in vitro* to stimulate myosin II motor activity and in some cases to promote myosin II polymerization into bipolar thick filaments [11]–[13]. The importance of Rok in regulating RLC phosphorylation *in vivo* has been demonstrated using a phospho-mimic RLC in which amino acids 20 and 21 have been changed to glutamates. Expression of RLCE20E21 can rescue larval lethality in Rok mutant flies [14]. In addition, phosphorylation of RLC in pupal wing cells is Rok-dependent [14]. In *C. elegans*, Rok controls the rate of furrow contraction, and its function is antagonized by a phosphatase specific for the RLC [15]. Thus, clearly the RLC is an important target for Rok in regulating contractile events both *in vitro* and *in vivo*.

Rok activation may lead to RLC phosphorylation stimulating the myosin II motor to affect force production and cleavage during cytokinesis. However, we recently showed that both Rho1 and Rok are not only essential for cell cleavage, but also for the initial accumulation of myosin II at the cleavage furrow of *Drosophila* S2 cells [1], suggesting that phosphorylation of the RLC may be involved in the recruitment of myosin II to the furrow. There is precedent for RLC phosphorylation affecting myosin II localization to the cell cortex. Royou et al. showed that expression of RLCE20E21 could restore myosin localization to the cortex of Rok-inhibited embryos during axial expansion [16], and Chodagam, et al. reported that expression of RLC-E20E21 restores the cell-cycle dependent recruitment of myosin II to the cortex of *Drosophila* embryos in mutants of CP190, a protein that interacts with centrosomes during mitosis and binds to microtubules *in vitro* [17]. However, Jordan and Karess reported that non-phosphorylatable RLC is correctly localized in *Drosophila* egg chambers [9], suggesting that different cellular events involving myosin II may be regulated differently. Thus, we wanted to test specifically for the importance of RLC phosphorylation in the localization of myosin II to the equatorial cortex of cells during mitosis, where together with actin and other proteins it forms a cytokinetic ring. Furthermore, we wanted to assess the relative importance of Rok-induced phosphorylation of myosin II regulatory light chain in myosin II localization and cytokinesis compared to the phosphorylation of other known Rok substrates, such as PTEN [18], Lim Kinase [19], and ERM proteins [20], [21]. We show here that phosphorylation of the RLC is required for myosin II localization to the equatorial cortex during mitosis and that the essential role of Rok in myosin II localization and for cytokinesis is to maintain phosphorylation of the myosin RLC.

## Results

### RLC phosphorylation is required for myosin II recruitment to the cleavage furrow

**Figure 1:**
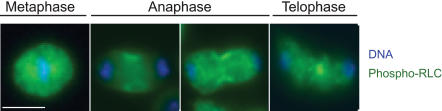
Myosin II RLC is phosphorylated in the cleavage furrow of dividing *Drosophila* cells. Immunofluorescent localization of DΝΑ (blue), and phospho-RLC (green) in S2 cells. The scale bar = 5 µm.

While phospho-RLC has been detected in the cytokinetic furrows of mammalian cells [22], [23], to our knowledge its presence has not been verified in the furrows of dividing *Drosophila* cells. Thus, we first assayed for the presence of phosphorylated RLC in dividing *Drosophila* S2 cells by staining these cells with an antibody specific for T20S21-phosphorylated RLC [24]. Phospho-RLC was detected at the equatorial cortex from early anaphase through telophase (Figure 1).

**Figure 2:**
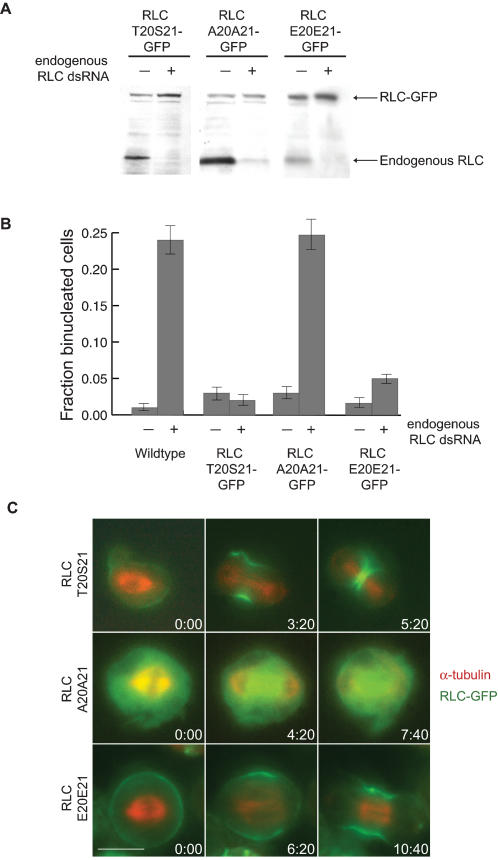
Phosphorylation of the RLC is necessary for cytokinesis and myosin II recruitment to the cleavage furrow. **(A)** Expression of mutant RLC constructs with alternative codons allows for depletion of only the endogenous RLC by RNAi. Cells expressing RLC-GFP constructs were treated with dsRNA that targets the endogenous sequence for 5 days. Proteins in the cell lysates of treated and untreated cells were separated by SDS-PAGE and immunoblotting with an anti-RLC antibody revealed that the lower molecular weight endogenous RLC is specifically depleted while the higher molecular weight GFP chimera is still expressed. **(B)** Phosphorylation of the RLC is essential for cytokinesis. S2 cells were treated with dsRNAs for 5 days to deplete the endogenous RLC and the fraction of approximately 100 cells that were binucleated (mean±SE) as measured by DAPI staining was calculated. The bar graphs show fraction of binucleate cells with and without treatment with RNAi against native RLC for wild type cells, cells expressing wildtype RLCT20S21-GFP, non-phosphorylatable RLCA20A21-GFP, and phospho-mimic RLCE20E21-GFP. **(C)** Phosphorylation of the RLC is essential for myosin II recruitment to the cleavage furrow. S2 cells expressing RLC-GFP chimeras (green) and RFP-α-tubulin (red) were depleted of the endogenous RLC by RNAi treatment for five days, and timelapse fluorescence microscopy was used to film cells as they attempted to divide. Both RLCT20S21-GFP (Row 1) and RLCE20E21-GFP (Row 3) were recruited to the equatorial cortex during mitosis and the cells contracted. However, RLCA20A21-GFP (Row 2) was not recruited to the equatorial cortex at anaphase and the cells did not contract. The scale bar = 5 µm.

We then designed three constructs to test whether phosphorylation of the RLC is necessary for myosin II recruitment to the cleavage furrow. In *Drosophila* embryos RLCA20A21 and RLCE20E21 have been shown to function as non-phosphorylatable and phospho-mimic mutants respectively [14], [16]. We therefore designed a wildtype construct RLCT20S21-GFP, a non-phosphorylatable construct RLCA20A21-GFP, and a phospho-mimic construct RLCE20E21-GFP in which 300 base pairs in the coding region of each RLC gene sequence were replaced with non-endogenous codons to allow for the specific depletion of the endogenous RLC using dsRNA designed against the endogenous codon sequence (Figure S1). Depletion of the endogenous RLC is necessary because of the filamentous nature of functional myosin II motors. In the presence of endogenous RLC, the mutant proteins will co-assemble with the endogenous RLCs onto myosin heavy chains resulting in myosin II filaments that contain a mixture of wildtype and mutant RLCs. Figure 2A shows that RNAi was successful in depleting only the endogenous RLC and allowing for expression of the mutant-GFP chimeric proteins. Dilution series experiments confirmed that the endogenous RLC was expressed at less than 5% control levels (data not shown).

In wild type cells, depletion of RLC results in a cytokinesis defect, as measured by an increase in multinucleated cells (Figure 2B). Expression of the GFP-tagged wild type version of the regulatory light chain, RLCT20S21-GFP, in cells depleted of the endogenous RLC rescued the cytokinesis defect (Figure 2B), and RLCT20S21-GFP was recruited normally to the cleavage furrow (Figure 2C, Movie S1). However, expression of the RLCA20A21-GFP non-phosphorylatable protein did not rescue the cytokinesis defect caused by depletion of the endogenous RLC (Figure 2B). This would be expected simply because activation of the myosin II motor domain by phosphorylation of the RLC is expected to be required for contraction of the furrow. Therefore, to test specifically for myosin II localization defects we used live-cell microscopy to film cells that express both the RLC-GFP constructs and mRFP-α-tubulin as the cells transitioned into anaphase and cytokinesis. Importantly, in the presence of the endogenous RLC, expression of RLCA20A21-GFP does not cause a cytokinesis defect (Figure 2B) and RLCA20A21-GFP is properly recruited to the equatorial cortex (data not shown). This demonstrates that the RLCA20A21-GFP is properly folded, associates with the myosin II heavy chain, and presumably is part of myosin bipolar thick filaments that contain both wild type and mutant forms of the RLC. Furthermore, this demonstrates that RLCA20A21-GFP does not cause a dominant negative effect on cytokinesis. After depletion of the endogenous RLC, however, RLCA20A21-GFP is not recruited to the equatorial cortex, and the cells do not elongate at anaphase nor contract (Figure 2C, Movie S2), similar to cells depleted of myosin II heavy chain, Rho1, or Rok [1], [25]. Approximately two-thirds of these cells filmed exhibited membrane ruffling during anaphase as shown in Figure 2C and Movie S2. These data suggest that phosphorylation of the RLC is necessary for recruitment of myosin II to the cleavage furrow of dividing *Drosophila* cells.

On the contrary, expression of the phospho-mimic RLCE20E21-GFP does not induce a cytokinesis defect upon depletion of the endogenous RLC (Figure 2B). In addition, the phospho-mimic RLCE20E21-GFP is properly recruited to the equatorial cortex, and the cells furrow normally after depletion of the endogenous RLC (Figure 2C, Movie S3). Despite some variations in the example cells shown in Figure 2C, examination of many cells of each type showed that the RLCT20S21-GFP expressing cells and the RLCE20E21-GFP expressing cells show no significant difference in the timing between anaphase onset and furrowing onset (p = 0.15), the amount of time it takes to complete furrowing (p = 0.10), nor the distribution of RLC-GFP during cytokinesis. However, the phospho-mimic RLCE20E21-GFP accumulates in the cortex during interphase and metaphase to a greater extent than the wildtype RLCT20S21-GFP (see, for example, Figure 2C).

### The only essential role of Rho Kinase in cytokinesis is the maintenance of RLC phosphorylation

**Figure 3:**
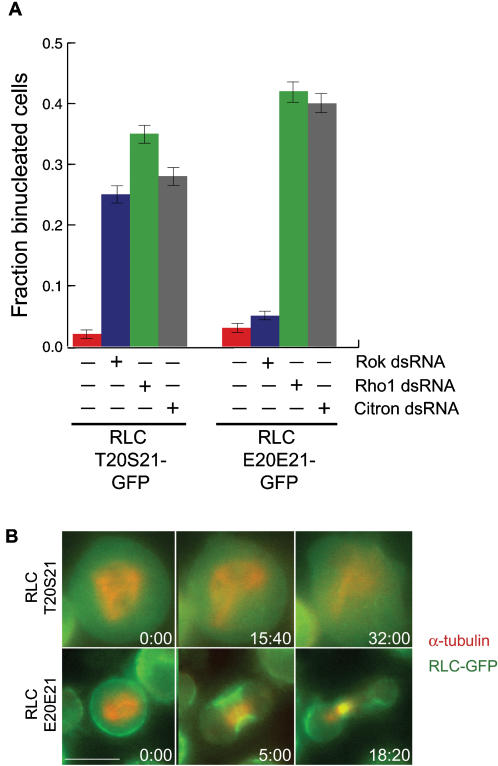
The only essential role of Rok during cytokinesis is to maintain phosphorylation of myosin II RLC. **(A)** S2 cells expressing RLCT20S21-GFP or RLCE20E21-GFP were treated with dsRNAs for 5 days to deplete Rho1, Rok, or Citron and then fixed and stained with DAPI. The fraction of binucleated cells (mean, ±SE for approximately 200 cells) was determined by fluorescence microscopy. Expression of RLCE20E21-GFP rescues depletion of Rok, but not Rho1 or Citron. **(B)** Expression of phospho-mimic RLC rescues Rok depletion. S2 cells expressing RLC-GFP chimeras (green) and RFP-α-tubulin (red) were depleted of the Rok by RNAi treatment for five days, and timelapse fluorescence microscopy was used to film cells as they attempted to divide. RLCT20S21-GFP (Row 1) was not recruited to the equatorial cortex at anaphase and the cells did not contract. RLCE20E21-GFP (Row 2) was recruited to the equatorial cortex during mitosis and the cells contracted. The scale bar = 5 µm.

In order to test whether Rok plays essential multifaceted roles in myosin II localization, we tested whether expression of phospho-mimic RLCE20E21-GFP could rescue Rok depletion. As a control, RLCT20S21-GFP expressing S2 cells showed a significant cytokinesis defect upon Rok depletion (Figure 3A) and also showed a significant failure to localize the myosin to the equatorial cortex of the mitotic cell (Figure 3B, Movie S4). Expression of the phospho-mimic RLCE20E21-GFP, however, rescued the cytokinesis defect in Rok depleted cells (Figure 3A). In addition, cells depleted of Rok but expressing RLCE20E21-GFP recruited myosin II normally to the equatorial cortex during mitosis (Figure 3B, Movie S5). Thus, despite its many potential targets, the sole essential role of Rok for myosin II localization is to maintain phosphorylation of the RLC. RLCE20E21-GFP was not able to rescue Rho1 depletion (Figure 3A), consistent with data showing that Rho1 activates multiple proteins essential for cytokinesis besides Rok, including Diaphanous [26] and Citron [27].

### Citron Kinase is not essential for RLC phosphorylation during cytokinesis

**Figure 4:**
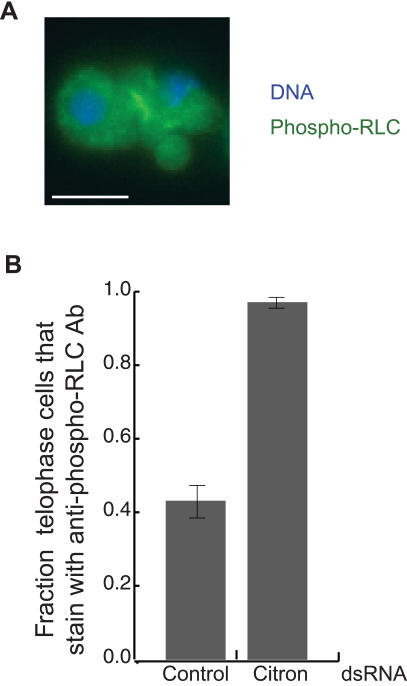
Citron is not essential for RLC phosphorylation. **(A)** Immunofluorescent localization of DNΑ (blue) and phospho-RLC (green) in Citron depleted cells. Notice the membrane blebs in the telophase furrow as well as the anti-phospho-RLC antibody staining in the midbody. Scale bar = 5 µm. **(B)** Depletion of Citron probably affects the structure of telophase midbodies. S2 cells were depleted of Citron by treatment with RNAi for 5 days and then fixed and stained with an anti-phospho-RLC antibody. Late telophase cells were scored for antibody staining (mean, ±SE for 30 cells). While less than half of all control cells were stained, nearly all Citron depleted cells were stained.

Both Rok and Citron are activated by Rho1, are found in cleavage furrows, and have been shown to phosphorylate the RLC [5]–[7]. However, while Rho1 and Rok depletion induce an early cytokinesis failure, Citron depletion results only in a late cytokinetic defect in which membrane blebs form in the telophase midbody [28]–[31]. Surprisingly, expression of RLCE20E21-GFP does not rescue the cytokinesis defect caused by Citron depletion in S2 cells (Figure 3A), and phospho-RLC is found at the cleavage furrow of Citron depleted cells late in cytokinesis, when one might have suspected Citron to be needed to keep the RLC phosphorylated (Figure 4). Previous studies showed that expression of RLC-E20E21 could rescue the rough eye phenotype of *Drosophila* Citron mutants indicating an *in vivo* role for Citron in RLC phosphorylation [28]. However, our results show that phosphorylation of the RLC is not the essential role of Citron during cytokinesis of S2 cells, again emphasizing that different cellular events involving myosin II may be regulated somewhat differently.

A different potential role for Citron is suggested by our observations of antibody staining in late telophase cells. We found that in cells that express RLC-GFP [32], RLC-GFP is found at the midbody of all late telophase cells indicating myosin II is present there in all late telophase cells. However, only 43% and 37% of these cells stain with the anti-phospho-RLC antibody or an anti-myosin II heavy chain antibody [33] respectively (n = 30 cells). Therefore, antibody binding must be inhibited in some late telophase cells probably due to a structural change in late furrows. Interestingly, we found that upon depletion of Citron, anti-phospho-RLC antibody staining was found in nearly all late telophase S2 cells (97%, n = 30 cells) (Figure 4). The increase in antibody staining in Citron depleted cells as compared to normal cells suggests that Citron depletion results in a structural change in late furrows that allows for antibodies to bind that are somewhat excluded from normal furrows. Thus, Citron may be important for maintaining the structural integrity of the late midbody.

## Discussion

We have shown that myosin II RLC localized at the cleavage furrow is phosphorylated and that this phosphorylation is required for the proper recruitment of myosin II to the equatorial cortex. While such a role was suggested by earlier studies showing that Rok is essential for myosin II localization to the equatorial cortex of dividing *Drosophila* S2 cells [1], this is the first direct demonstration that the RLC phosphorylation state regulates proper recruitment of myosin II to the equatorial cortex.

Cells expressing a phospho-mimic RLC did not show a cytokinesis defect. Thus, dephosphorylation of the RLC at late telophase must not be required to complete cytokinesis. In addition, localized regulation of RLC phosphorylation at the furrow must not be necessary for myosin II recruitment. Instead, pre-“phosphorylated” myosin II can be recruited to the furrow. Thus, the widely-held view that myosin II accumulates at the equatorial cortex because of cortical flow is most likely incorrect. According to the cortical flow model, localized activation of the myosin II motor at the equatorial cortex induces tension there such that the contents of the cortex flow toward the center of the cell resulting in the accumulation of cleavage furrow proteins. However, phospho-mimic RLC should be equally active throughout the cortex, and yet it accumulates at the equator. Also arguing against the cortical flow model is our observation that F-actin is not required for myosin II recruitment [1].

Interestingly, we have shown that the essential role of Rok in cytokinesis is simply to maintain phosphorylation of myosin II RLC, as shown by the rescue of all Rok depletion cytokinesis phenotypes by expression of phospho-mimic RLC. This is especially interesting because Rok has also been shown to phosphorylate the lipid phosphatase PTEN [18] which plays a role in cytokinesis in *Dictyostelium* [34] and Lim kinase which phosphorylates Cofilin [35], an essential cytokinesis protein [36]. Rok can contribute to myosin II RLC phosphorylation in two ways: through the phosphorylation of the RLC and by suppressing the activity of the myosin phosphatase by phosphorylation of its subunit Mbs [10]. Our results are consistent with Rok being involved in either role. However, Hickson et al. have shown that Rok is important for triggering the onset of furrowing even in the absence of Mbs [25]. In addition, depletion of any other known myosin light chain kinase in combination with Rok and Mbs depletion does not induce a furrowing defect [25]. Therefore, it is likely that Rok is involved in myosin localization during mitosis by directly phosphorylating the RLC.

Surprisingly, we found that Citron Kinase is neither required for RLC phosphorylation during cytokinesis nor can phospho-mimic RLC rescue the cytokinesis defect induced by Citron depletion. Instead our results suggest that Citron may be important for maintaining the structural integrity of the late midbody. A role for Citron in maintaining the structure of late furrows is consistent with the phenotype of Citron mutants in which the membrane of late furrows blebs dramatically [28]–[31]. Previously, phosphorylation of the RLC has been hypothesized to be important for this role by regulating the interaction of the RLC with anillin, a protein that binds F-actin, membranes, and phospho-RLC myosin II [28]. However, because phospho-mimic RLC cannot rescue Citron depletion, the role of Citron in late furrows is not likely to be RLC phosphorylation.

This study clearly demonstrates a role for RLC phosphorylation in regulating myosin II localization in *Drosophila*. Further studies should help elucidate the molecular mechanism by which cells differentiate between phospho- and non-phospho-RLC myosin II and specifically recruit the former to the equatorial cortex of a cell in mitosis. While phospho-RLC myosin II has a higher motor activity, this activity is not likely to be involved in regulating myosin II recruitment to the furrow because F-actin is not required for this recruitment [1]. Interestingly, bipolar thick filament formation of some myosin IIs is regulated by RLC phosphorylation, and studies in *Dictyostelium* have clearly demonstrated that formation of bipolar thick filaments is required for myosin II localization to the furrow in that system [37]. Intriguingly, phospho-mimic RLC- containing myosin II appears to be over-accumulated in the cortex of interphase and metaphase cells in a similar manner to *Dictyostelium* myosin II that is constitutively assembled into thick filaments [37], [38]. Further studies will be needed to show whether thick filament formation plays a role in myosin II localization in cells of higher eukaryotes.

## Materials and Methods

### Plasmid Construction and Cell Culture

Plasmids for expression of RLC-GFP constructs were created as follows: Plasmid pCasper-sqh-sqh-gfp [39] was digested with SpeI and NotI restriction enzymes to release a fragment of the RLC gene (called *sqh*) encoding the phosphorylation sites. The fragment was subcloned into pBluescript (Stratagene) and Quikchange Mutagenesis (Stratagene) was used to mutate the sites. The fragment was then subcloned back into the digested pCasper-sqh-sqh-gfp plasmid to create plasmids pCasper-sqhA20A21-gfp and pCasper-sqhE20E21-gfp. The alternative codon sequence fragment of DNA was synthesized by DNA 2.0 (Menlo Park, CA) and contained a 5′ SrfI site and a 3′ AgeI site. The synthesized fragments contained the coding region of the *sqh* gene from P82 to the end of the gene at Q174 as well as five amino acids from the linker region between *sqh* and *gfp* (see Supporting Information, Figure S1). pCasper-sqh-gfp constructs were digested with SrfI and AgeI restriction enzymes and the synthesized fragments were ligated into the plasmids to create plasmids pCasper-sqhT20S21alt-gfp, pCasper-sqhA20A21alt-gfp, and pCasper-sqhE20E21alt-gfp.

*Drosophila* S2 cells were maintained on plates in Schneider's *Drosophila* Media (Gibco) supplemented with 10% heat-inactivated FBS at 27°C. The RFP-α-tubulin/RLC-GFP stable cell lines were created by co-transfection of plasmid pUbp-mRFP-α-tubulin [1] and pCasper-Sqhalt-GFP plasmids using Cellfectin Reagent (Invitrogen). Stably transfected cells were selected with 1 mg/ml G418 (Life Technologies) for three weeks before removal of drug from the culture.

### RNA Interference

Approximately 5×10^5^ cells were plated in individual wells of 24-well tissue culture plates and treated with 10 µg of dsRNA for five days, as previously described [40]. Templates for transcription of dsRNA were generated by PCR with the T7 promoter sequence upstream of the following sequences: RLC (5′CATCAACTTCACCATGTTCC3′ and 5′TTACTGCTCATCCTTGTCC3′); Rok (5′GAGAACACTCAAAAGCTGAAAAAG3′ and ACAGTTCCTTCTGTAGCTGGTTTT3′); Rho1 (5′TTTGTTTTGTGTTTAGTTCGGC3′ and 5′ATCAAGAACAACCAGAACATCG3′); Citron (5′TTCAACCTCTGGAAGTTATCGG3′ and 5′GTGAAACCGTTGGTGATATGC3′)

### Immunostaining and Fluorescence Microscopy

S2 cells were fixed in 3% formaldehyde and permeabilized in 0.1% Triton X-100 in PBS. Cells were stained with 1 µg/mL DAPI (Molecular Probes) to visualize DNA as well as with the following primary antibodies: 1∶1000 Dm1α anti-α-tubulin (Sigma), 1∶500 anti-phospho-RLC [24], and 1∶500 anti-myosin II heavy chain [33]. For live cell imaging, cells were plated in cell imaging chambers (Applied Scientific) and allowed to settle on the coverslips for 30 minutes. Images were taken with a Zeiss Axiovert 200 inverted epifluorescence microscope equipped with a 100× objective lens. Images were taken every 20 seconds.

### Immunoblotting

S2 cells were lysed and protein levels were quantified using Bradford Reagent (BioRad). Dilution series of control cell lysates as well as RNAi treated cell lysates were loaded on each gel and separated by SDS-PAGE electrophoresis. Proteins were transferred to nitrocellulose membranes, the membranes were blocked in 5% skim milk, and probed with 1∶2500 dilution of an anti-RLC antibody provided by Tsien Hsu (Medical University of South Carolina), followed by 1∶5000 dilution of goat-anti-rabbit-IgG-HRP antibody (Pierce Biochemicals). Signals were developed with ECL reagent (Amersham) and quantified using ImageQuant (Amersham).

## Supporting Information

Figure S1 The endogenous and alternative sequences for the RLC in pCasper-sqh-gfp constructs. In black is the endogenous sequence of the RLC from pCasper-sqh-gfp from P82 to Q174 and into the linker region between sqh and gfp. In red is the sequence consructed with alternative codons. Each base that has been changed is highlighted in yellow. The SrfI and AgeI sites used in cloning are marked above the sequences.
(3.65 MB TIF)

Movie S1 RLCT20S21-GFP is localized to the cleavage furrow. Drosophila S2 cells expressing RLCT20S21-GFP (green) and mRFP-alpha-tubulin (red) were treated with dsRNA for 5 days to deplete the endogenous RLC. Epifluorescence microscopy was used to film cells as they transitioned from metaphase into anaphase and telophase. Images were taken at 20 second intervals and movies are played at 10 frames/second. Figure 2C, row 1 contains images from this movie.
(0.27 MB MOV)

Movie S2 RLCA20A21-GFP is not localized to the equatorial cortex and cells do not contract. Drosophila S2 cells expressing RLCA20A21-GFP (green) and mRFP-alpha-tubulin (red) were treated with dsRNA for 5 days to deplete the endogenous RLC. Epifluorescence microscopy was used to film cells as they transitioned from metaphase into anaphase and telophase. Images were taken at 20 second intervals and movies are played at 10 frames/second. Figure 2C, row 2 contains images from this movie.
(0.59 MB MOV)

Movie S3 RLCE20E21-GFP is localized to the cleavage furrow. Drosophila S2 cells expressing RLCE20E21-GFP (green) and mRFP-alpha-tubulin (red) were treated with dsRNA for 5 days to deplete the endogenous RLC. Epifluorescence microscopy was used to film cells as they transitioned from metaphase into anaphase and telophase. Images were taken at 20 second intervals and movies are played at 10 frames/second. Figure 2C, row 3 contains images from this movie.
(0.36 MB MOV)

Movie S4 Wildtype RLC-T20S21-GFP is not localized to the cleavage furrow after Rok depletion. Drosophila S2 cells expressing RLCT20S21-GFP (green) and mRFP-alpha-tubulin (red) were treated with dsRNA for 5 days to deplete Rok. Epifluorescence microscopy was used to film cells as they transitioned from metaphase into anaphase and telophase. Images were taken at 20 second intervals and movies are played at 10 frames/second. Figure 3B, row 1 contains images from this movie.
(5.85 MB AVI)

Movie S5 Phospho-mimic RLCE20E21-GFP is localized to the cleavage furrow even after Rok depletion. Drosophila S2 cells expressing RLCE20E21-GFP (green) and mRFP-alpha-tubulin (red) were treated with dsRNA for 5 days to deplete Rok. Epifluorescence microscopy was used to film cells as they transitioned from metaphase into anaphase and telophase. Images were taken at 20 second intervals and movies are played at 10 frames/second. Figure 3B, row 2 contains images from this movie.
(3.12 MB AVI)
