# Supplementary figures and images for: Rho Kinase's Role in Myosin Recruitment to the Equatorial Cortex of Mitotic Drosophila S2 Cells Is for Myosin Regulatory Light Chain Phosphorylation
Source: PLoS One. 2006 Dec 27;1(1):e131. doi: 10.1371/journal.pone.0000131 (PMC1762308; doi:10.1371/journal.pone.0000131)

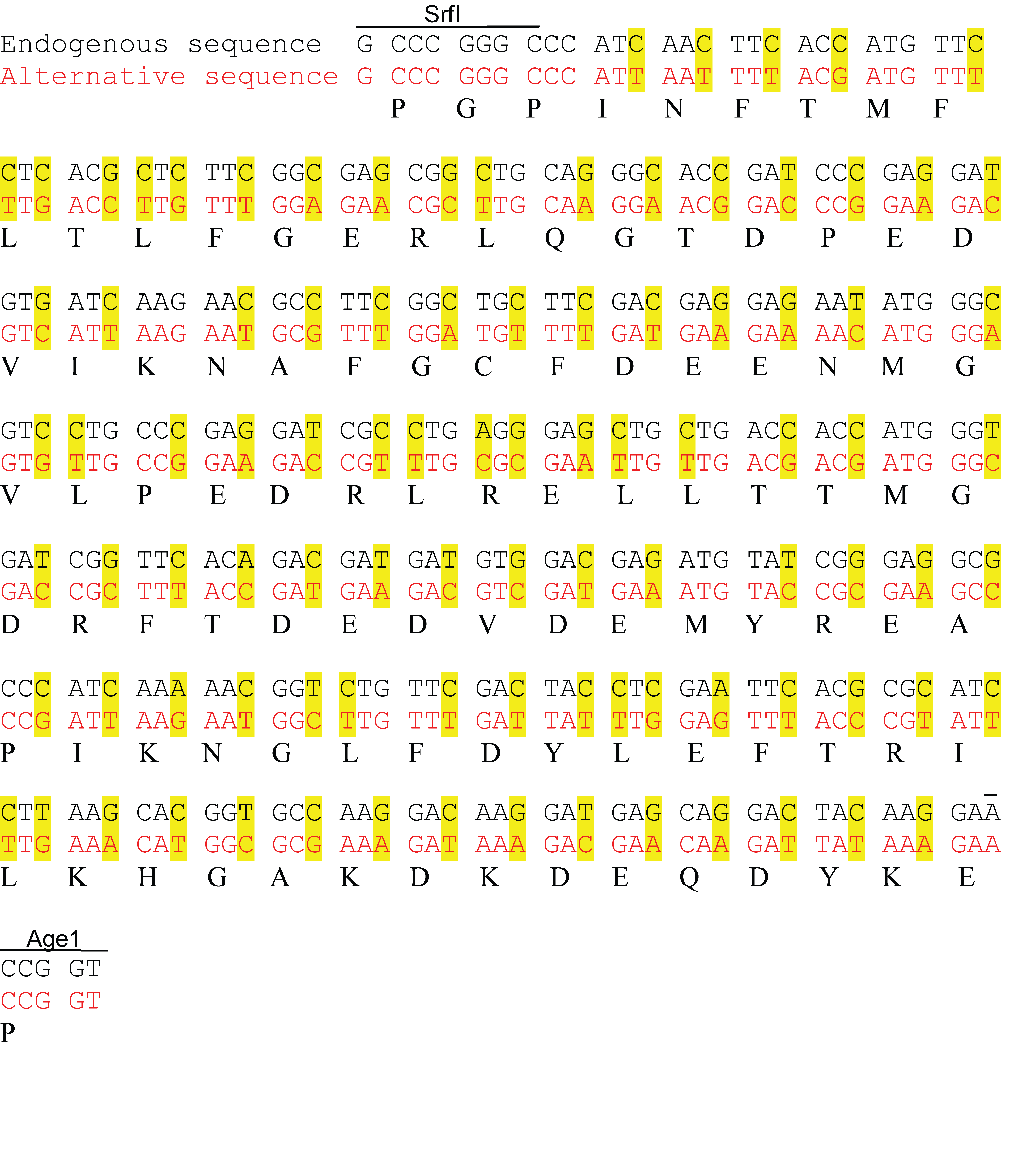

Supplement: Figure S1 — The endogenous and alternative sequences for the RLC in pCasper-sqh-gfp constructs. In black is the endogenous sequence of the RLC from pCasper-sqh-gfp from P82 to Q174 and into the linker region between sqh and gfp. In red is the sequence consructed with alternative codons. Each base that has been changed is highlighted in yellow. The SrfI and AgeI sites used in cloning are marked above the sequences. (3.65 MB TIF) [file pone.0000131.s001.tif]
